# Tempering Instruments for Cutting

**Published:** 1864-06

**Authors:** Charles Butler


### TEMPERING INSTRUMENTS FOR CUTTING.

BY CHARLES BUTLER.

In order to be able to make first class operations, it is of the highest importance that we should have instruments that are adapted to the work we have daily coming before us as dental surgeons. We are often called upon to bring into requisition the skill of the artizan as well as the artist; *both* being indispensable in order to render the highest expression of our science.

It is not possible for a man that *simply* manufactures instruments for the market to know their adaptability, as one that is constantly operating, and often finds it a hard matter to make the case fit the instruments, instead of having the instruments well adapted to the case in hand.

Now, in order to obviate this difficulty that so often arises, especially to those that have more recently entered the field of operative dentistry, and are somewhat removed from the large cities, where instruments can be more readily pointed by a manufacturer, under the direction of the operator, as circumstances may require, I would suggest a simple remedy for such inconveniences.

First, procure some *square* steel of the best quality, of such sizes as you need for the various cutting instruments required. The best English steel can be procured of Jessop & Sons, 91 John St., N. Y. Three sizes are sufficient for all cutting instruments. One one-fourth inch, and the next two sizes smaller. Round steel is unfit for cutting instruments. After the steel has been carefully forged, and the points properly formed, taking care not to over-heat the point at *any* part of the process of working it up, I have found the following material a great assistant in producing good cutting points as a recarbonizer: Rosin, 1 lb.; Lamp Black, 2 oz.; Pulverized Charcoal, ½ oz. Melt them together in a tin can or box, and it is ready for use.

Now heat the point up carefully to a dull red, then dip it in the mixture a moment; heat again carefully up to a red and plunge in soft water, or the following as a tempering water: Sal Ammoniac, 4 oz.; Nitrate of Potash, 4 oz.; Indigo, 1 oz.; Rock Salt, 1 lb.; Soft water, 2 gals. Keep it corked in a stone jug. The point being hard, it should be grasped with pliers, supporting the shaft at the same time; then hold the shank of the instrument over the flame of the lamp, or throw a fine blaze on the shank with the blow pipe, which, perhaps, is better drawing the shank down to a spring temper to the angle, or as far as you wish a cutting blade.

If you wish to cut enamel, the temper should not be started at all in the blade; leave it hard, and if it has not been over-heated in working it will not break readily. Even for cutting dentine the point may be left HARD. All blades should be left thick and ground into shape or an edge after being tempered. "By so doing you will not be likely to burn the point while hardening."—C. Palmer, Warren, Ohio. Plugging instruments can be worked and tempered by the above process, by drawing them down to a spring temper.

Great care must be taken to clean and polish the instrument after using the tempering water, or they will rust badly. This is one great objection, and I only use it for large instruments—water being sufficient for all ordinary purposes; but the carbonizing material I consider of much value in producing good instruments.

